# Prevalence of stroke in children admitted with sickle cell anaemia to Mulago Hospital

**DOI:** 10.1186/s12883-016-0704-2

**Published:** 2016-09-17

**Authors:** Deogratias Munube, Elly Katabira, Grace Ndeezi, Moses Joloba, Samden Lhatoo, Martha Sajatovic, James K. Tumwine

**Affiliations:** 1 Department of Paediatrics and Child Health, School of Medicine, College of Health Sciences, Makerere University, P. O. Box 7072, Kampala, Uganda; 2 Department of Internal Medicine, School of Medicine, College of Health Sciences, Makerere University, P. O. Box 7072, Kampala, Uganda; 3 Deparment of Microbiology, School of Biomedical Sciences, College of Health Sciences, Makerere University, P.O. Box 7072, Kampala, Uganda; 4 Department of Neurology, University Hospitals - Case Medical Centre, 111000 Euclid Avenue, Cleveland, USA; 5 Neurological and Behavioural Outcomes Centre, University Hospitals – Case Medical Centre, 10524 Euclid Avenue, Cleveland, USA

**Keywords:** Sickle cell anaemia, Stroke, Children, Uganda, Sub-Saharan Africa

## Abstract

**Background:**

Stroke is a major complication of sickle cell anaemia (SCA). It occurs commonly in childhood with about 10 % of children with sickle cell anaemia getting affected by this complication. In Uganda, there is paucity of data on the prevalence of stroke in children admitted in a tertiary institution. We determined the prevalence of stroke amongst children with SCA admitted to Mulago National Referral Hospital in Uganda and described the ir co-morbidities.

**Methods:**

We conducted a retrospective record review of children with SCA admitted from August 2012 to August 2014 to the Paediatric Haematology Ward of Mulago Hospital in Kampala, Uganda. The target population was SCA children age 6 months-17 years of age. A descriptive analysis was used to summarize the demographic characteristics and clinical diagnosis.

**Results:**

There were 2,176 children with SCA admitted who were included in this study. There were 147 children with stroke. Their mean age 6.1, (SD 3), with a male to female ratio was 1:1 (71 males and 76 females). The M: F ratio of non-stroke children was 1.1:1 (1084 males and 945 females) with a mean age of 5.2, (SD 3). The prevalence of stroke was 6.8 % (147 of 2176). Amongst the children with stroke, 72.1 % (106 of 147) had co-morbidities which included severe anaemia 21.7 % (23 of 106), bacteraemia and vaso-occlusive crisis 17 % (18 of 106), pneumonia 8.4 % (9 of 106) and malaria 6.6 % (7 of 106).

**Conclusion:**

The prevalence of stroke in hospitalized Ugandan children with SCA was 6.8 %. Children with stroke were often admitted with other medical conditions such as severe anaemia, bacteraemia and vaso-occlusion.

**Electronic supplementary material:**

The online version of this article (doi:10.1186/s12883-016-0704-2) contains supplementary material, which is available to authorized users.

## Background

Sickle cell anaemia (SCA) is a common genetic condition due to a haemoglobin (Hb) disorder inheritance of mutant haemoglobin genes from both parents. It is estimated that 30 million persons are affected with SCA [1]. It is a major health problem and the most common inheritable disease in Africa. Approximately 60 % of individuals with SCA live in Sub Saharan Africa. Each year about 300,000 infants are born with major haemoglobin disorders- including more than 200,000 cases of SCA in Africa [1].

Stroke as a complication of SCA affects 6 to 17 % of children and young adults worldwide [2]. The risk of stroke is highest during the first decade of life and it is most significant between the ages of 2 and 5 years [3]. Approximately 10 % of patients will have a stroke by the age of 20 [3]. Stroke subtypes vary by age in SCD patients. The incidence of the ischaemic stroke, which constitutes 54 % of all strokes in SCA, is highest during the first decade of life and after age 30. Haemorrhagic stroke is more common among individuals in their 20s [2].

A child with SCA has a stroke risk that is 333 times greater than that of a healthy child without SCA or heart disease [3]. The landmark STOP trial by Adams et al. in 1998 found that primary stroke can be prevented by chronic transfusions after identifying children at risk using a transcranial Doppler (TCD) screening of the anterior or middle cerebral arteries [4]. As a result of this finding, the National Heart, Lung and Blood Institute recommend that TCD screening must be used as a modality to identify children at risk for stroke [5]. Subsequent stroke incidence studies by Fullerton et al. in the USA which have assessed the impact of chronic blood transfusion in children who have been identified by TCD screening have shown a decline in annual rates of stroke [6]. In Uganda TCD screening is not available for the population of children with SCA.

Despite SCA being common in Uganda [7], data is lacking regarding the prevalence of stroke in children with SCA admitted. We conducted a retrospective chart review of children admitted with SCA and determined the prevalence of stroke. In addition, we described the medical co-morbidities in the same children with stroke in Mulago Hospital, Kampala.

## Methods

### Overview

This was a retrospective study, conducted at Mulago Hospital, the main National Referral and Teaching Hospital for Makerere University College of Health Sciences in Kampala, Uganda. A single reviewer extracted all the records of all children with SCA aged <18 years admitted to the Haematology Ward of Mulago Hospital during the study period from August 2012 to August 2014. In Uganda, there is no policy for routine sickle cell testing such as newborn screening. Children with symptoms and signs of sickle cell disease are tested. The confirmation of SCA was by Hb electrophoresis. Some of the children would have been tested during the infancy period when signs of SCD emerge and others are tested based on their clinical presentation to the hospital. In addition, some children were tested for SCD based on the presentation of a stroke. A standardized data collection sheet was used to collect information on demographic characteristics (name, age, sex, address) and clinical diagnosis, which included information on the diagnosis and other medical conditions in these children on admission. The data was cleaned to identify any duplication of patients. In Mulago Hospital, each patient is given a unique admission number and on subsequent admissions the same number is issued. In addition, the old file of the patient was retrieved were possible to include the new details of the admission. The first stroke admission was included in the analysis. Repeated admissions of the same patient with stroke were excluded. In cases where the admission number was not indicated these cases were also excluded.

### Setting

In Uganda, Mulago Hospital is the National Referral hospital where patients with SCA are referred from other peripheral health facilities.. In the Acute care unit (ACU) of the paediatric department, we admit 7 to 10 children with sickle cell disease per day for an acute crisis. They are initially stabilized in the ACU and later transferred on a daily basis to the haematology ward of the hospital. In addition, the hospital has a SCA day care clinic which cares for the children with SCA. The clinic’s average attendance is 50 to 70 clients per day. They receive their routine medications and some interventions such as blood transfusions, intravenous antibiotics, and parenteral analgesics. Hydroxyurea is not registered for use in Uganda for people living with SCA.

Stroke management in Mulago Hospital: Children with SCA who are admitted with a clinical diagnosis of stroke are given a simple transfusion to raise the haemoglobin level up to 10 g/dl. Imaging such as CT scan and MRI/MRA are not accessible because of the cost associated with the tests. All radiological investigations are paid for at a cost to the patients in the hospital. The hospital only has a single CT scan machine and does not have an MRI machine. All cases of stroke are diagnosed using clinical symptoms and signs. As a result, the attending physician/paediatrician depends on their clinical skills to determine whether the patient has an ishaemic or haemorraghic stroke. In the event that the brain imaging is available, it usually is done well after 24 to 48 h of admission to the hospital. The children who suffer a stroke are offered a chronic transfusion program which involves a monthly visit to the sickle cell clinic for review and a simple blood transfusion. However, the biggest challenge is the lack of a reliable supply of blood to the clinic. This is because of the high demand for blood products to treat other conditions such as severe malaria anaemia, haematological malignancies, road traffic accidents and obstetric emergencies in the hospital and country at large.

### Operational definitions

#### Primary diagnosis

This was considered as the first problem registered by the clinician on the admission record.

#### Secondary diagnosis

This was considered as the second problem registered by the clinician on the admission record.

#### Severe anaemia

This was defined using the World Health Organization classification as a haemoglobin concentration of less than 7 g/dl [8].

#### Stroke

This was defined using the World Health Organization definition of stroke: “rapidly developing clinical signs of focal (or global) disturbance of cerebral function, with symptoms lasting 24 h or longer or leading to death with no apparent cause other than of vascular origin [9].

### Study variables

Data was extracted from the hospital medical record books using a standardized questionnaire. The information included socio-demographic characteristics such as: age, sex, tribe, address and clinical diagnosis such as primary and secondary diagnoses where applicable. The diagnosis extracted was based on the decision made by the attending team on discharge of the patient. In the event more than one diagnosis was made, we sub-divided them into a primary diagnosis and a secondary diagnosis.

### Data analysis

All data was entered into Microsoft excel and analysis was done using the same software. The prevalence was calculated as the proportion of observation units that ever exhibited the state of interest, including those units that already exhibited the state during the period of study. Data was analyzed using descriptive statistics including proportions and percentages.

## Results

**Fig. 1:**
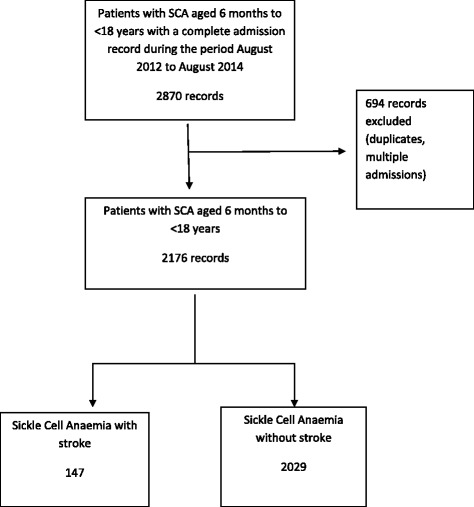
Study profile of children admitted with sickle cell anaemia

A total of 2,870 records were extracted. Of which 2,176 records were included in the analysis as shown in Fig. 1. All children with stroke were diagnosed by clinical symptoms and signs using the WHO definition of a clinical stroke. The male to female ratio of children with stroke was 1:1 (71 males, 76 females). The M: F ratio of non-stroke children was 1.1:1 (1084 males and 945 females). A total of 147 patients had a diagnosis of stroke. The prevalence of stroke was 6.8 % (147 of 2176). The mean age of patients with stroke was 6.1 years (SD 3). The mean age of non-stroke children was 5.2, (SD 3). The age distribution of patients with stroke showed that 91.1 % (134 of147) of children were less than 10 years of age. The age group of greater than 5 to 10 years comprised the largest percentage of 44.9 % (66 of 147) as shown in Table 1. The age distribution of the non-stroke patients is shown in Table 1. Amongst the 147 patients with stroke, 27.9 % (41 of 147) presented with a stroke as a primary diagnosis, while 72.1 % (106 of 147) presented with stroke and a secondary diagnosis. The common co-morbidities included were severe anaemia, 21.7 % (23 of106); bacteraemia, 17 % (18 of 106); and vaso-occlusive crisis, 17 % (18 of 106). Other co-morbidities are shown in Table 2. The major reason for admission for children with SCA without stroke was a vaso-occlusive crisis. The rest of the other causes are shown in Table 3. Among the children with stroke, all were managed with simple transfusions to raise their Hb level up to 10 g/dl according to the department of paediatrics sickle cell stroke protocol. It should be noted that only four children with stroke had been started on hydroxurea. All the children had been started on the medication after suffering a primary or recurrent stroke.

**Table 1:** Baseline characteristics of stroke and non-stroke children with sickle cell anaemia

Variable	Stroke, *N* = 147 (%)	Non-stroke, *N* = 2029 (%)
Age (years)		
0–2	9(6.1)	483(23.8)
> 2–5	59(40.1)	652(32.1)
> 5–10	66 (44.9)	685(33.8)
> 10–18	13(8.8)	209(10.3)
Gender		
Male	71 (48.2)	1084 (53.4)
Female	76 (51.9)	945 (46.6)
Ethnicity^a^		
Ganda^b^	97 (65.9)	1,346 (66.3)
Others	50 (34.1)	683 (33.7)
Residence		
Urban	121(82)	1136 (56)
Rural	26 (18)	893 (44)

^a^Local tribes of Uganda. ^b^ Ganda the most common tribe in the central region of Uganda

where Mulago Hospital is located

**Table 2:** Co-morbid conditions in children with sickle cell anaemia stroke

Co-morbid diagnosis	No of patients *N* = 106	Frequency (%)
Severe anaemia (Hb <7 g/dl)	23	21.7
Vaso-occlusive crises	18	17.0
Bacteraemia	18	17.0
Pneumonia	9	8.4
Malaria	7	6.6
Acute chest syndrome	3	2.8
Acute watery diarrhea	2	1.9
Others^a^	5	4.7

^a^acute diarrhea, severe malnutrition, cellulitis, dental infections, urinary tract infection

**Table 3:** Reasons for admission of the children with sickle cell anaemia without stroke

Diagnosis	No of patients, *N* = 2029	Frequency (%)
Vaso-occlusive crises	811	40.0
Severe anaemia (Hb <7 g/dl)	503	24.8
Pneumonia	197	9.7
Bacteraemia	194	9.5
Acute chest syndrome	84	4.1
Malaria	78	3.8
Dactylitis	66	3.2
Hyperhaemolytic crisis	25	1.2
Osteomyelitis	14	0.7
Others^a^	62	3.1

^a^Tuberculosis, Meningitis, Cholecystitis, Urinary tract infections, leg ulcers, priapism, paediatric HIV, pyelonephritis, abdominal malignancy, acute watery diarrhea, febrile convulsions, URTI (frequency of less than five)

## Discussion

In this study, we have determined the prevalence of stroke in children admitted in the haematology ward of Mulago Hospital. We found that the prevalence of stroke in children admitted to our paediatric haematology ward was 6.8 %. This is similar to other studies done in other parts of Africa such as Cameroun and Nigeria [10, 11]. It is also similar to the earlier reported prevalence in North America before the introduction of interventions which have led to a decrease in the prevalence of stroke in the developed world [3, 12].

There was an equal male to female ratio in our study population. This was in contrast to a study by Balkaran et al. in Jamaica which showed a higher incidence of stroke in boys [12].

The majority of children with SCA and stroke in our sample were between the ages 2 to 10 years. This is similar to other reports by Ohene-Frempong et al. in the USA and George et al. in Nigeria where they documented the occurrence of stroke in children with SCA in the age bracket of 2 to 10 years [3, 13]. We also noted that the majority of our children were aged 5 to 10. This contrasts with studies by Ohene-Frempong et al. in Philadelphia and Balkaran et al. in Jamaica who both followed up children from infancy up to childhood. They reported an earlier occurrence between the ages of 2 to 5 years of age for stroke in their populations [3, 12]. This difference could be explained by a healthy survivor effect. Most children in Uganda with SCA die by their fifth birthday [14]. Those who survive by natural selection or who have more access to care and treatment develop their complications at a later period in their lifetime.

In this study, we found that 72.1 % of our children with stroke had co-morbidities. The common co-morbidities included severe anaemia, bacteraemia and vaso-occlusive crisis. This was similar to studies by Kehinde et al. and Amayo et al. [11, 15]. They noted that in their respective centers, children who presented with a stroke had other infections or conditions at the time of presentation. In 1978, Powars et al. found that children with severe anaemia and bacteraemia where at a higher risk for stroke [2]. Currently, in North America these conditions are not as common because of the introduction of pneumococcal vaccination. In Uganda, we still have high incidences of severe bacterial infections due to lack of appropriate preventative interventions [16]. There is poor access to penicillin prophylaxis, lack of access to pneumococcal vaccination for children with sickle cell disease (SCD) and also lack of dedicated sickle cell clinic’s outside of Mulago hospital to provide the necessary care to this high risk group. In Uganda, we have only one dedicated sickle cell clinic and day care centre which is located within Mulago National Referral Hospital. There is no transcranial Doppler screening program in the country for children with sickle cell anaemia.

We describe for the first time the prevalence of stroke in our population of children with SCA. We also describe some co-morbid conditions that the children with stroke may present with. The knowledge of the prevalence of stroke in our population of children with SCA will strengthen the need to provide comprehensive care for children with SCA. This will include early identification of children at risk for stroke using transcranial Doppler screening and the provision of chronic transfusion for those at risk to prevent stroke occurrence [17]. The burden of SCD in Uganda is high with an estimated 245,000 babies born with SCD per year [7]. With such a large population and our prevalence rate of 6.8 %. We postulate that the introduction of a TCD program in Uganda will prevent 8,000 new stroke cases per year.

There are several strengths to this retrospective study. There were a large number of children with sickle cell anaemia who were admitted to Mulago hospital during the study period. The haematology ward is where the children with SCA are admitted. It was established in 2012 as a means of improving care of children with haematological disorders. Prior to that year, the children were admitted all over the existing wards of the paediatric directorate. Mulago Hospital is a national referral centre and thus most children with complications of sickle cell disease such as stroke would be referred to the hospital.

This study has some limitations. This was a retrospective study which relied on already existing patient records. We selected only those charts that had all the information required for abstraction. This being a hospital based study; we acknowledge that not all children with sickle cell stroke will be admitted to Mulago hospital being a tertiary unit in an urban area. In addition, not all children with SCA would be cared for at this facility. In Uganda, there is no established national ambulance service for referral of patients from all areas of the country. This may prevent the referral of other children with stroke to the facility. However, the findings are a representative of children with SCA presenting to a national referral center in Uganda.

## Conclusions

In conclusion, the prevalence of stroke in admitted children with SCA was 6.8 % in Uganda. Co-morbidity was a common finding in these children. We recommend introduction of trans-cranial Doppler screening in Uganda as a modality to identify those who may be at risk of stroke to prevent its occurrence. This study is part of a larger sickle cell anaemia stroke study which is looking to identify risk factors for stroke in children with SCA in Mulago Hospital. These results have led the department of paediatrics and child health at Mulago Hospital to plan for the introduction of transcranial Doppler screening in the hospital. In addition, future community based studies should be carried out to determine the incidence of stroke in children with SCA.

## Additional file

Additional file 1: The supplementary file is an excel sheet entitled SCA prevalence study primary data. It contains the primary data for the sickle cell anemia patients. This includes data on the age, sex and diagnoses of the patients on admission. (XLS 215 kb)

## Abbreviations

ACU: Acute care unit
CT: Computer tomography
Hb: Haemoglobin
MRI: Magnetic resonance imaging
SCA: Sickle cell anaemia
SCD: Sickle cell disease
TCD: Transcranial Doppler
USA: United States of America
